# Thrombophilia Screening in Young Patients With Cryptogenic Ischemic Stroke

**DOI:** 10.1161/STROKEAHA.125.053251

**Published:** 2026-03-16

**Authors:** Sofie Abels, Maximillian Sihvo, Liisa Tomppo, Shakar Kutal, Nicolas Martinez-Majander, Lauri Tulkki, Tomi Sarkanen, Pekka Jäkälä, Petra Redfors, Juha Huhtakangas, Pauli Ylikotila, Bettina von Sarnowski, Nilufer Yesilot, Ulrike Waje-Andreassen, Ana Catarina Fonseca, Patricia Martinez Sanchez, Janika Kõrv, Phillip Ferdinand, Kristina Ryliskiene, Alessandro Pezzini, Radim Licenik, Marialuisa Zedde, Mika Lehto, Juha Sinisalo, Eva Gerdts, Hugo Ten Cate, Tuukka A. Helin, Lotta Joutsi-Korhonen, Tímea Szántó, Frank-Erik de Leeuw, Jukka Putaala

**Affiliations:** Department of Neurology (S.A., M.S., L. Tomppo, S.K., N.M.-M., L. Tulkki, J.P.), Helsinki University Hospital and University of Helsinki, Finland.; Department of Cardiology, Heart and Lung Center (M.L., J.S.), Helsinki University Hospital and University of Helsinki, Finland.; Department of Clinical Chemistry, HUS Diagnostic Center (T.A.H., L.J.-K.), Helsinki University Hospital and University of Helsinki, Finland.; Department of Neurology (S.A.), Radboud University Medical Center, Nijmegen, the Netherlands.; Radboud Institute for Medical Research and Innovation, Donders Institute for Brain, Cognition and Behaviour, Centre for Neuroscience (F.-E.d.L.), Radboud University Medical Center, Nijmegen, the Netherlands.; Department of Neurology, Tampere University Hospital, Wellbeing Services County of Pirkanmaa, and Faculty of Medicine and Health Technology, Tampere University, Finland (T. Sarkanen).; Department of Neurology, Neurocenter Neurology, Kuopio University Hospital, Finland (P.J.).; Department of Neurology, Sahlgrenska University Hospital and Department of Clinical Neuroscience, Institute of Neuroscience and Physiology, Sahlgrenska Academy at University of Gothenburg, Sweden (P.R.).; Department of Neurology, Oulu University Hospital and University of Oulu, Finland (J.H.).; Department of Neurology, Turku University Hospital and University of Turku, Finland (P.Y.).; Department of Neurology, University Medicine Greifswald, Germany (B.v.S.).; Istanbul Faculty of Medicine, Department of Neurology, Istanbul University, Turkey (N.Y.).; Department of Neurology, Haukeland University Hospital, Bergen, Norway (U.W.-A.).; Faculty of Medicine, Department of Neurology, Hospital de Santa Maria, University of Lisbon, Portugal (A.C.F.).; Department of Neurology, Torrecardenas University Hospital, University of Almeríaa, Spain (P.M.S.).; Department of Neurology and Neurosurgery, University of Tartu, Estonia (J.K.).; Neurosciences, University Hospitals of North Midlands NHS Trust, Stoke-on-Trent, United Kingdom (P.F.).; Faculty of Medicine, Center of Neurology, Vilnius University, Lithuania (K.R.).; Department of Medicine and Surgery, Department of Emergency, University of Parma and Stroke Care Program, Parma University Hospital, Italy (A.P.).; North West Anglia NHS Foundation Trust, Acute Stroke Centre, Peterborough, United Kingdom (R.L.).; Neurology Unit, Stroke Unit, Azienda Unità Sanitaria Locale-IRCCS Reggio EmiliaSanitaria Locale-IRCCS Reggio Emilia, Italy (M.Z.).; Department of Clinical Science, Center for Research on Cardiac Disease in Women, University of Bergen, Norway (E.G.).; Department of Internal Medicine, Maastricht University Medical Center and CARIM School for Cardiovascular Diseases, the Netherlands (H.T.C.).; Unit of Coagulation Disorders, Department of Hematology, Helsinki University Hospital Comprehensive Cancer Center and University of Helsinki, Finland (T. Szántó).; University of Eastern Finland, Kuopio (P.J.).

**Keywords:** adult, case-control studies, ischemic stroke, risk factors, stroke, thrombophilia

## Abstract

**BACKGROUND:**

The incidence of cryptogenic ischemic stroke (CIS) in young adults is increasing, particularly among those without traditional vascular risk factors. Thrombophilia may contribute to CIS pathogenesis, yet guidelines differ on the relevance of screening. We investigated the sex-specific prevalence of routinely applied thrombophilia screening in young-onset CIS and its association with clinical characteristics and standard laboratory results.

**METHODS:**

We included young patients with CIS aged 18 to 49 years from the SECRETO study (Searching for Explanations for Cryptogenic Stroke in the Young: Revealing the Triggers, Causes, and Outcome). Routinely used thrombophilia panels obtained at admission and repeated testing within 1 year of stroke were analyzed. Ten thrombophilia markers were assessed and categorized according to the grade of deviation into lowest, low, and high thrombosis risks. Factors associated with any deviation in thrombophilia markers were investigated with multivariable logistic regression.

**RESULTS:**

Of 598 initially enrolled patients with CIS, 556 undergoing baseline thrombophilia testing were analyzed (median age, 41.0 [interquartile range, 34.1–45.8] years; male:female ratio 1.2:1). Of these, 120 (21.6%) and 36 (6.5%) were retested by 3 and 12 months, respectively. At baseline, any thrombophilia abnormality was observed in 206 patients (37.1%; men, 38.2%; women, 35.7%). High-risk thrombophilia was identified in 29 patients (5.2%) at baseline, in 13 retested patients (11.1%) at 3 months, and in 5 retested patients (13.9%) at 12 months. Overall, 45 patients (8.1%) had persistent thrombophilia abnormalities (men, 7.9%; women, 8.3%). Physical inactivity, a history of venous thromboembolism, low HDL (high-density lipoprotein)-cholesterol, and low hemoglobin were associated with any deviation in the thrombophilia markers. Anticoagulation was initiated in 50.0% of patients with abnormal baseline thrombophilia versus 35.7% without (*P*=0.042) and in 26.9% versus 6.2% with and without persistent abnormalities (*P*<0.001).

**CONCLUSIONS:**

Over a third of young adults with CIS had thrombophilia deviations at admission although high-risk thrombophilia results remained rare. Screening may be considered in young patients with CIS with a history of venous thromboembolism, physical inactivity, and low hemoglobin or HDL-cholesterol.

Stroke is the leading cause of death and long-term disability worldwide, affecting over 15 million people annually, including at least 1.5 million young adults (aged <50 years).^1^ The incidence of ischemic stroke in these young adults is rising, primarily driven by cryptogenic ischemic stroke (CIS).^2^ Up to 50% of early onset strokes are cryptogenic or associated with findings for which determining causality is often challenging, such as patent foramen ovale (PFO).^3^

Traditional vascular risk factors, such as hypertension, smoking, and diabetes, have been recognized as important contributors to early onset ischemic stroke.^4^ However, many young patients lack these risk factors, suggesting a greater influential role of nontraditional factors, including venous thromboembolism (VTE), autoimmune disease, and thrombophilia, as supported by a recent retrospective case-control study and additional meta-analyses, underscoring the need for further investigation of these factors.^4–8^

Current knowledge on the impact of thrombophilia in young patients mainly derives from small-sized studies; larger studies assessing the prevalence of hypercoagulability findings specifically in young-onset patients with CIS are lacking. Thrombophilia screening panels used in patients with stroke are typically adopted from protocols used in VTE and include tests for both inherited and acquired thrombophilias, including factor V Leiden, prothrombin G20210A mutation, factor VIII activity, antithrombin activity, protein C activity, and free protein S antigen, as well as antiphospholipid antibodies, with some variability between laboratories.^9^ Abnormal levels of these markers are well-established risk factors for VTE, but their role in arterial thrombotic events is less clearly defined.^5,10^ The net result of thrombophilia is increased thrombin generation contributing to ischemic stroke risk through mechanisms such as paradoxical embolism from the venous side via PFO and, potentially, as a driver of the formation and progression of atherosclerotic lesions.^5,8,9,11^ Despite these hypotheses, routine thrombophilia screening in young patients with stroke is not currently recommended in clinical guidelines.^8,9,12–14^

We hypothesized that thrombophilia may play a significant role in the pathogenesis of CIS in young patients and be a frequent finding. To investigate this, we conducted a prospective international multicenter study to assess the prevalence and rate of thrombophilia retesting in young-onset patients with CIS. Furthermore, we assessed whether demographic factors or clinical and routine laboratory markers are associated with abnormal thrombophilia testing results.

## Methods

### Data Availability Statement

The data supporting the findings of this study are not publicly available but are available upon reasonable request and with permission of the SECRETO consortium (Searching for Explanations for Cryptogenic Stroke in the Young: Revealing the Triggers, Causes, and Outcome).

### Cohort Description

We collected detailed clinical and laboratory data from 598 patients aged 18 to 50 years with a first-ever CIS enrolled across 19 European centers between November 2013 and January 2022 in the SECRETO study.^3^ Ethical approval for the study was granted by local committees, and all participants provided written informed consent.

The main inclusion criteria were (1) age between 18 and 49 years at stroke onset and (2) hospitalization for first-ever, imaging-positive CIS, following a complete and timely minimum diagnostic work-up. The main exclusion criteria are (1) failure to obtain the minimum baseline tests within the first week following stroke onset and (2) completion of other baseline tests within the first 2 weeks after stroke onset.^3^

For the present analysis, we excluded patients with completely missing data on thrombophilia testing and those with missing data in >5 of the 10 assessed thrombophilia markers. Patients with established antiphospholipid syndrome before the index CIS were considered causally relevant and were, therefore, not enrolled.^3^

### Stroke Classification

CIS was classified according to the ASCO system (A: atherosclerosis, S: small vessel disease, C: cardiac source, and O: other source): either the absence of a detectable cause (grade 0) or an uncertain causality (grade II) or unlikely to be a direct cause (grade III), based on the most reliable diagnostic evidence available. Minor adaptations to these criteria were made to more accurately reflect the diagnostic uncertainty frequently encountered in young patients.^15^

### Definition of Risk Factors

Baseline data on lifestyle, psychosocial factors, and medical history were gathered using standardized questionnaires.^3^ Anthropometric measurements, blood pressure, and heart rate were obtained using standard protocols. Clinical history was retrieved according to the INTERSTROKE study (Global and Regional Effects of Potentially Modifiable Risk Factors Associated With Acute Stroke in 32 Countries), including cardiovascular disease, diabetes, dyslipidemia, hypertension, obstructive sleep apnea, abdominal obesity, current smoking, heavy alcohol use, unhealthy diet, physical inactivity, psychosocial stress, and depression.^16^ Nontraditional risk factors were defined as the following: history of inflammatory bowel disease, history of chronic kidney disease, history of chronic liver disease, history of autoimmune disease, history of hematologic disease or known thrombophilia, history of VTE, history of malignancy, and history of migraine with aura and illicit drug use within the past 12 months. Female-sex–specific risk factors included a history of gestational diabetes, gestational hypertension, eclampsia, pregnancy or puerperium, and current use of estrogen (Table S1). The presence of PFO was recorded and classified as high-risk if an atrial septal aneurysm or large right-to-left shunt was detected.^3^

### Follow-Up Visits

Patients attended a follow-up visit at 3 months and a call at 12 months after the index stroke, where medical and laboratory records were reviewed.

### Standard Laboratory Assessments

We analyzed a range of standard laboratory tests taken on admission, with known interplay with or potential impact on blood coagulation. These tests included CRP (C-reactive protein), hemoglobin, platelet count, leukocyte count, total cholesterol, LDL (low-density lipoprotein)-cholesterol, HDL (high-density lipoprotein)-cholesterol, triglycerides, ALAT (alanine aminotransferase), GGT (gamma-glutamyl transferase), creatinine, glucose, and international normalized ratio. Results were analyzed both as continuous and categorical variables. Accepted sex-specific cutoffs were applied for hemoglobin, ALAT, and GGT. HDL-cholesterol values below 0.9 mmol/L were considered low, according to Framingham study criteria.^17^ Most participating hospitals used CRP measurements with a lower cutoff indicating normal range, and to generate a continuous CRP variable, we imputed values below the cutoff (eg, <10 mg/L) using the midpoint between 0 and the cutoff.

### Thrombophilia Assessments

We recorded the performance and results of a total of 10 thrombophilia markers, including antithrombin activity, protein C activity, free protein S antigen, factor VIII activity, factor II 20210 mutation, factor V Leiden mutation, anticardiolipin antibodies, anti-β2-glycoprotein antibodies, lupus anticoagulant, and homocysteine concentration.

The study protocol recommended using a thrombophilia screening panel as provided by the local laboratory although the composition of these panels varied between participating sites.

Abnormal values reflecting higher thrombosis risk were determined according to the combination of accepted reference ranges, relevant literature, and consultation with coagulation specialists (T.S. and H.T.C.) and specialists of clinical laboratory medicine (T.A.H. and L.J.-K.), acknowledging that no universally accepted cutoffs exist.^17–21^ Based on the reference ranges of these sources, the level of each continuous thrombophilia test result was categorized as normal, low risk, and high risk. Heterozygous factor II 20210 and factor V Leiden mutations, anti-β2-glycoprotein antibodies or anticardiolipin antibodies between 10 and 40 U/mL, antithrombin activity of 60% to 79%, factor VIII activity of 150% to 189%, free protein S antigen of 40% to 70% for men or 40% to 56% for women, homocysteine concentration of 16 to 100 μmol/L, and protein C activity of 60% to 69% were classified as low-risk thrombophilia findings. Homozygous factor II 20210 and factor V Leiden mutations, high anti-β2-glycoprotein antibodies or anticardiolipin antibodies (>40 U/mL), antithrombin activity <60%, factor VIII activity >190%, free protein S antigen <40%, severe hyperhomocysteinemia (>100 μmol/L), protein C activity <60%, the presence of high titer of triple-positive antiphospholipid antibodies, or lupus anticoagulant, were classified as high-risk thrombophilia findings. The markers were further divided into 2 groups: inherited and acquired thrombophilia markers. Specific cutoff values for each marker are listed in Table S2.

Performance and results of thrombophilia testing were recorded over the 12 months of follow-up. Based on the overall thrombophilia results, we categorized patients into 4 thrombosis risk categories: lowest risk (all results within the normal range), low risk (at least 1 low-risk result but no high-risk results), high risk (at least 1 high-risk result), and combined risk (the presence of both low- and high-risk results).

To evaluate whether infections could affect thrombophilia test results, patients were asked about any symptoms of infection at the time of taking blood samples to screen for thrombophilia using a structured questionnaire. Furthermore, data on current use of antiplatelet or anticoagulation therapy were recorded at 3- and 12-month follow-ups.

### Primary and Secondary Outcomes

The primary outcome was the presence of at least 1 abnormal thrombophilia marker in the panel. Secondary outcomes included the presence of high-risk thrombophilia results and the persistence of abnormality in thrombophilia markers assessed from repeated testing performed by 3 or 12 months of follow-up.

### Statistical Analyses

All statistical analyses were performed using SPSS, version 29.0, for Microsoft Windows.

Categorical variables are presented as n (%) with comparisons between groups performed using the Pearson χ^2^ and Fisher exact tests. Continuous variables with normal distributions are expressed as means and SDs and were compared using the independent sample *t* test. For nonnormally distributed continuous variables, data are presented as medians with interquartile range, and comparisons were made using the Mann-Whitney *U* test or the Kruskal-Wallis test, as appropriate.

Multivariable logistic regression analyses using the backward likelihood ratio selection method were conducted to identify independent predictors of primary and secondary outcomes, adjusting for age, sex, and other prespecified relevant factors. The models were constructed sequentially, with the first model including each independent predictor to estimate unadjusted odds ratios, the second including demographic variables (age and race), the third adding additionally clinical risk factors (a history of diabetes, a history of venous thrombosis, a history of chronic multisystem disorder, physical inactivity, and hypercholesterolemia), and the fourth model adding further incorporating standard laboratory values (low hemoglobin and low HDL-cholesterol). Dyslipidemia was excluded from the fourth model due to its collinearity with low HDL-cholesterol. C-statistics and receiver operating characteristic curves were provided for each model. In addition, sex-specific models were performed to explore potential sex differences in associations. We performed a sensitivity analysis assessing the robustness of the associations by repeating the logistic regression for the primary outcome by excluding patients with missing factor VIII and homocysteine values. A 2-sided *P*<0.05 was considered statistically significant in all analyses.

This study was reported according to the STROBE guidelines (Strengthening the Reporting of Observational Studies in Epidemiology; Supplemental Material).

### Ethical Approvals and Consents

All participating study centers obtained approvals from the appropriate local ethics committees, in accordance with relevant ethical and regulatory standards. Before enrollment, written informed consent was obtained from all participants.^3^

## Results

From the initial data set of 598 patients, 42 patients were excluded from the analysis: 22 patients with completely missing data on thrombophilia testing and an additional 20 patients with missing data for >5 of the 10 assessed thrombophilia markers. Therefore, a total of 556 patients with CIS were included in the final analysis (Figure S1).

Of the included 556 patients with CIS (median age, 41.0 [interquartile range, 34.1–45.8] years; male:female ratio 1.21:1), 94.4% were of White European ancestry. The most prevalent comorbidities were abdominal obesity, dyslipidemia, hypertension, and smoking. Half of the patients had a positive family history of stroke. Women showed a significantly higher prevalence of a history of autoimmune disease and migraine with aura compared with men. A clinically relevant PFO was detected in 38.5% of the patients (Table 1).

**Table 1.**
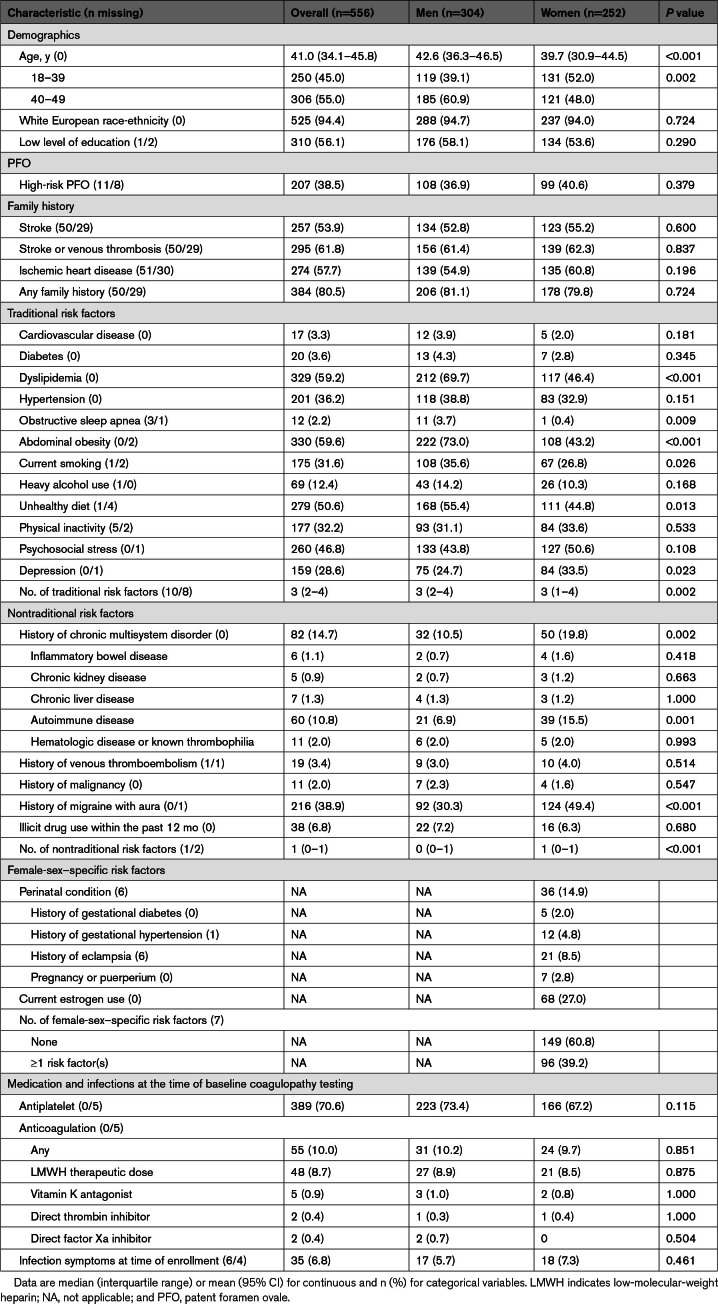
Clinical Characteristics of Young Patients With Cryptogenic Ischemic Stroke, Stratified by Sex (n=556)

### Standard Laboratory Results

In the baseline routine laboratory assessments, elevated CRP levels were observed in 4.7% of patients. Low hemoglobin levels were identified in 16.9%, while over one-third of patients presented with high total cholesterol. In addition, 50% of patients had elevated LDL-cholesterol levels. Regarding sex differences, women had lower hemoglobin concentrations and higher platelet counts. In contrast, men had higher levels of LDL-cholesterol and triglycerides, and lower levels of HDL-cholesterol. In addition, concentrations of ALAT, GGT, creatinine, and blood glucose were significantly higher in men (Table 2).

**Table 2.**
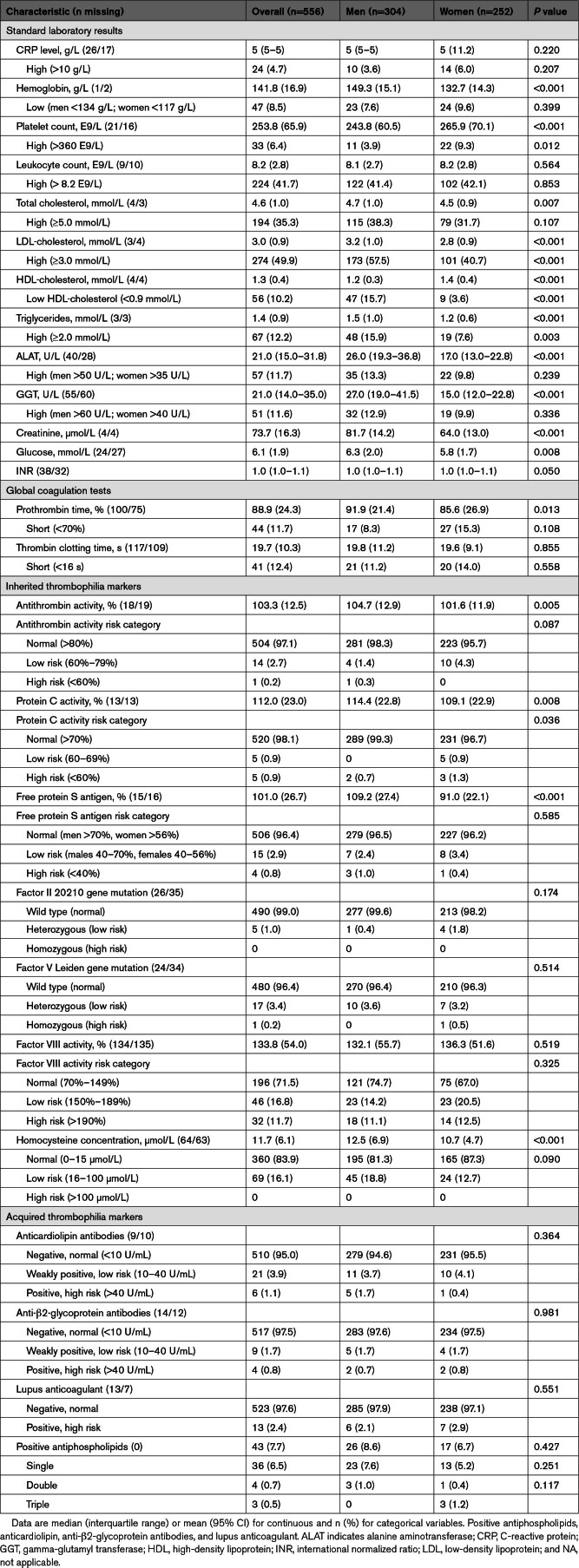
Baseline Results of Standard and Thrombophilia Laboratory Tests Among the Included Young Patients With Cryptogenic Ischemic Stroke, Stratified by Sex (n=556)

### Thrombophilia Screening

All included patients underwent thrombophilia screening at baseline, performed within 1 week after admission. Completeness of the screening varied across individual markers, with the highest proportions of missing appearing for homocysteine concentration (23.0%) and factor VIII activity (48.4%; Figure [A]).

**Figure.**
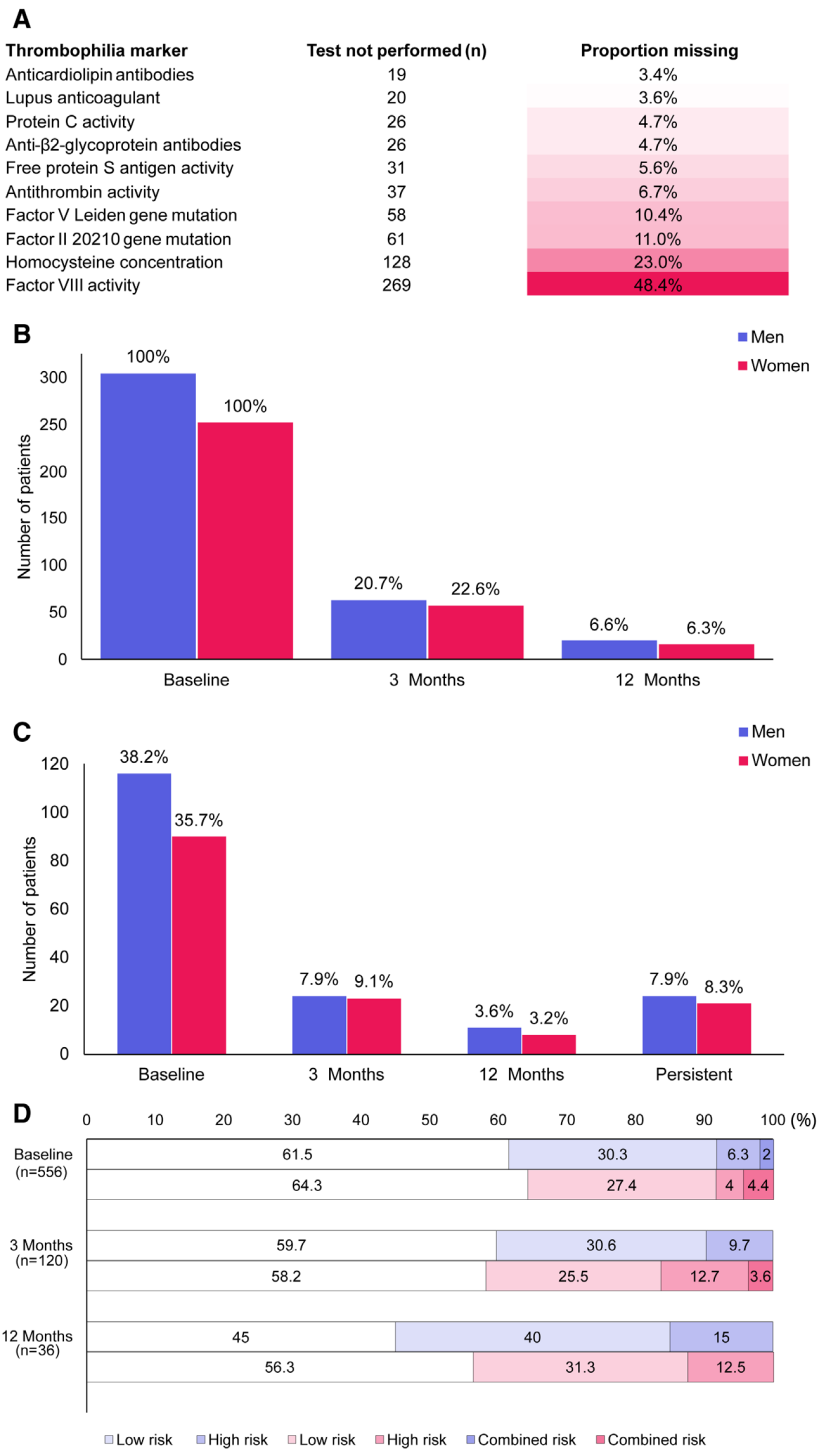
**Descriptive analysis of continuous thrombophilia markers at baseline, stratified by sex (n=556). A**, Thrombophilia markers not tested. **B**, Screening performance. **C**, Any abnormality in the thrombophilia panel. **D**, Clinical thrombosis risk (%).

Follow-up screening at 3 and 12 months was performed in 21.6% and 6.5% of patients, respectively, with no difference between sexes (Figure [B]).

Abnormalities in at least 1 of the 10 thrombophilia markers were detected in 37.1% of patients at baseline. Persistent abnormalities in at least 1 thrombophilia marker at 3 and 12 months were observed in 8.1%, with no difference between sexes (Figure [C]). Figure (D) depicts thrombosis risk categories at baseline and by 3 and 12 months among the tested patients, with Table S3 denoting further details. At baseline, 5.2% of patients were categorized as high thrombosis risk, while 11.1% and 13.9% of retested patients showed this risk at 3 and 12 months, respectively. In addition, 3.1% showed the combination of both low and high thrombosis risks, with no sex difference. Retesting at 3 months was not limited to patients with abnormal baseline coagulopathy panel results, and retesting at 12 months was not restricted to those with abnormal findings on the initial retest. Among patients with persistent abnormalities, 55.6% were low risk and 28.9% high risk, compared with 26.2% low risk and 3.1% high risk in those without persistent abnormalities (Table S4).

Regarding individual thrombophilia markers, homocysteine concentration, factor VIII activity, factor V Leiden gene mutation, and free protein S antigen most frequently showed abnormal results at baseline. Notably, retesting was infrequently performed, particularly for factor VIII activity and homocysteine concentration. Regarding sex differences, significantly higher levels of homocysteine were observed in men. Female patients, in turn, had significantly lower levels of antithrombin activity, protein C activity, and free protein S antigen (Table 2; Figure S2).

### Factors Associated With Thrombosis Risk

Patients in the category of any thrombosis risk were older and more often aged 40 to 49 years and more frequently of White European ancestry compared with those with normal thrombophilia test results at baseline. Furthermore, a higher prevalence of history of dyslipidemia, physical inactivity, history of chronic multisystem disorders, history of VTE, and low levels of HDL-cholesterol and hemoglobin were found in patients showing any thrombosis risk (Table 3). Head-to-head comparisons between thrombosis risk groups showed similar rates in the low-risk group compared with patients with the lowest risk (Table S5). Notably, a family history of stroke, VTE, or ischemic heart disease was more frequent in those with high-risk thrombophilia results (96.2%) compared with patients with normal (79.4%) and low-risk (78.5%) results.

**Table 3.**
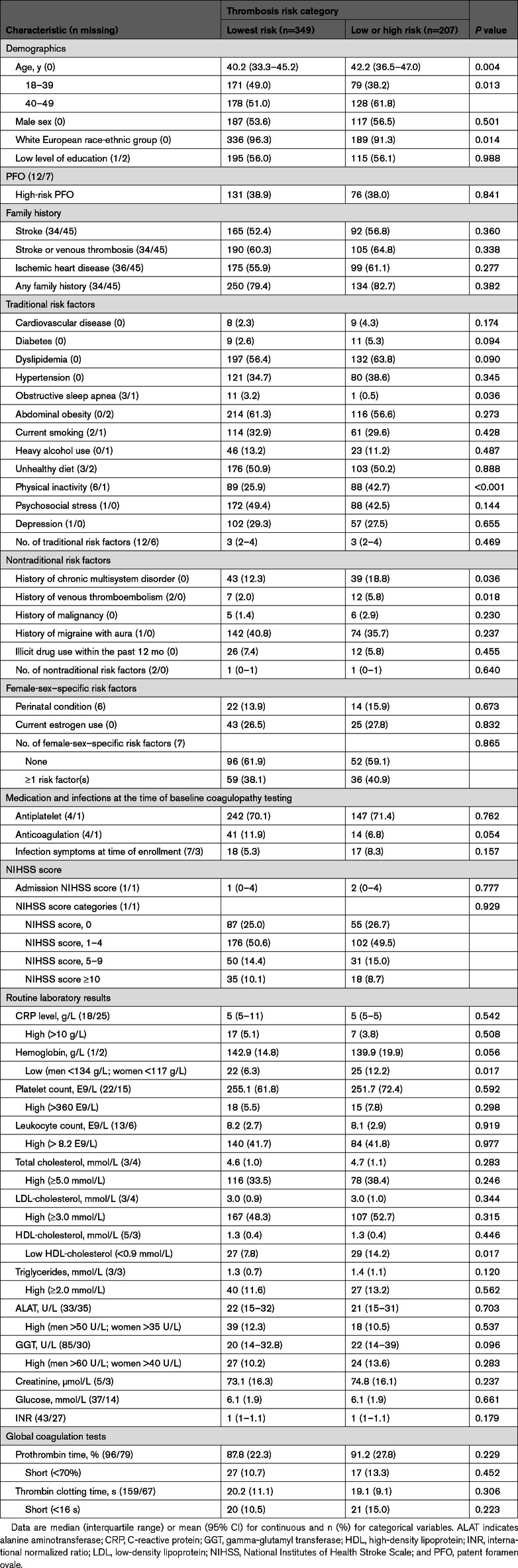
Comparison of Clinical Characteristics Between Patients With Lowest Thrombosis Risk (All Results Within the Normal Range) and Low or High Thrombosis Risk (at Least 1 Low-Risk Result or at Least 1 High-Risk Result but the Absence of the Combination of Both Low- and High-Risk Results; n=556)

In the first logistic regression model adjusted for demographic variables, both older age (40–49 versus 18–39 years) and White European ethnicity were significantly associated with the presence of any thrombophilia marker abnormality. After adding clinical risk factors in the second model, the effects of age and race-ethnicity were no longer statistically significant, whereas dyslipidemia, physical inactivity, and a history of VTE were associated with any thrombophilia marker abnormality. In the third model, a history of VTE, low HDL-cholesterol, low hemoglobin, and physical inactivity remained significantly associated with any thrombophilia marker abnormality (Table 4). C statistics and the corresponding receiver operating characteristic curves showing the area under the curve for each model appear in Figure S3.

**Table 4.**
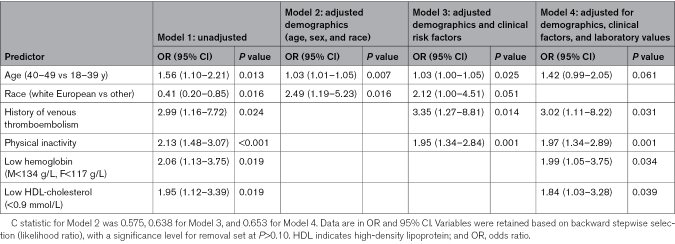
ORs and 95% CIs From Multivariable Logistic Regression Models for Primary Outcome Measure, Any Abnormal Findings in the Thrombophilia Panel

Logistic regression analysis assessing factors associated with high-risk thrombophilia findings (Table S6) did not reveal any statistically significant associations with demographics, comorbidities, and standard laboratory markers.

Sex-specific models showed a consistent association between physical inactivity and thrombosis risk (Tables S7 and S8). In addition, in the female-specific model, a history of VTE was associated with an increased thrombosis risk.

### Coagulopathy and PFO

Compared with patients with a high-risk PFO, baseline routine laboratory findings showed significantly higher prevalence of low hemoglobin, high platelet count, high leukocyte count, lower levels of HDL-cholesterol, and elevated liver enzymes among patients without a high-risk PFO. Regarding thrombophilia markers, higher levels of free protein S antigen and lower levels of factor VIII activity were found in patients with a high-risk PFO (Table S9; Figure S4).

### Initiation of Anticoagulation

Patients with abnormal thrombophilia results at baseline used anticoagulation significantly more frequently at 12 months than those without abnormal baseline results (50% versus 35.7%; *P*=0.042). Similarly, anticoagulation at 12 months was more frequent among patients with persistent abnormalities compared with those without persistent abnormalities (26.9% versus 6.2%; *P*<0.001). Patients receiving anticoagulation had a higher prevalence of high-risk thrombophilia compared with those not receiving anticoagulation (11.5% versus 4.6%; *P*=0.031). Changes in anticoagulation initiation from baseline to 3 and 12 months are illustrated in a Sankey diagram (Table S10; Figure S5).

### Sensitivity Analysis

To assess the robustness of the associations of the main regression model, we repeated the same logistic regression in a subset of patients without missing factor VIII and homocysteine data, resulting in a reduced sample size (n=245). In the second model, physical inactivity was statistically significantly associated with any deviation in the coagulopathy results. The third model showed a significant association between physical inactivity and low hemoglobin levels with any abnormal findings in the thrombophilia panel (Table S11).

## Discussion

The main findings of our study were a high overall frequency (37.1%) of abnormalities in the thrombophilia panel but a relatively low frequency (5.2%) of major high-risk deviations soon after young-onset CIS. Among patients undergoing retesting within 1 year of stroke, this proportion increased to 11.1% at 3 months and 13.9% at 12 months. Notably, only a subset of patients underwent retesting, that is, 21.6% and 6.5% at 3 and 12 months, respectively, reflecting the fact that retesting was at the discretion of the treating physician and local policies. We identified several clinical factors associated with abnormal thrombophilia findings, including a history of VTE, physical inactivity, and low levels of HDL-cholesterol or hemoglobin. Interestingly, we found no indication of more pronounced coagulopathy among patients with a high-risk PFO. Importantly, abnormal results at baseline or later time points appeared to affect anticoagulation initiation within 12 months of stroke, reflecting the clinical importance of coagulopathy screening in the young CIS population.

Our findings align with previous studies that identify thrombophilia as a relevant factor in young stroke populations.^22^ The associations with physical inactivity and prior VTE also align with existing evidence as contributors to prothrombotic states.^8,10,23^ Several clinical factors, namely, increasing age, history of VTE, physical inactivity, low hemoglobin, and low HDL-cholesterol, were independently associated with abnormal coagulopathy panel results in our study. These associations may reflect a convergence of prothrombotic and inflammatory pathways, forming a systemic thromboinflammatory milieu. Aging is known to be associated with a procoagulant shift in hemostasis, including increased levels of clotting factors and reduced fibrinolytic activity.^24^ A history of VTE is an expected correlate, as it may reflect underlying acquired or genetic thrombophilia, particularly at a young age.^8,10^ Physical inactivity may promote hypercoagulability via endothelial dysfunction, low-grade inflammation, and decreased fibrinolytic activity.^23^ The association of low hemoglobin may indicate underlying comorbid conditions, such as anemia-induced hypercoagulability, chronic inflammation, occult malignancy, or an undiagnosed autoimmune condition that can affect coagulation profiles.^25–27^ Low HDL-cholesterol has been linked to endothelial dysfunction and impaired antithrombotic capacity.^19^ In summary, these factors may contribute to a systemic environment favoring coagulation activation although their mechanisms may not all be identical. Some likely act synergistically, while others may reflect distinct biological processes that are captured by the same coagulopathy markers. In addition, baseline testing may be influenced by acute-phase reactions, potentially affecting thrombophilia markers when samples were taken shortly after the onset of stroke.

In our study, the discrepancy between baseline and repeated thrombophilia testing suggests selective retesting rather than a true shift in thrombosis risk. In comparison to other cohorts, including nonselected young ischemic patients with stroke, we reported higher numbers of elevated antiphospholipid and homocysteine levels, and a lower frequency of abnormalities in factor V Leiden mutation and antithrombin activity.^28–30^ Notably, laboratory data were collected from baseline in those studies, with retesting reported in only 1 study, and only performed when initial testing revealed abnormalities.^28^

Several sex differences were observed in the laboratory parameters, which are consistent with known sex-related variations in hematologic and metabolic parameters. Men exhibited higher levels of atherogenic lipids, homocysteine, and liver enzymes. In contrast, women showed lower hemoglobin levels, higher platelet counts, and lower levels of antithrombin activity, protein C activity, and free protein S antigen. These differences may suggest sex-specific pathogenetic thrombosis mechanisms of CIS.

Although findings in the literature are conflicting, with some studies supporting and others questioning the hypothesized mechanism of paradoxical embolism in PFO and the low prevalence of traditional stroke risk factors among patients with CIS with PFO,^8,30–32^ we anticipated a higher prevalence of thrombophilia marker abnormalities in patients with a high-risk PFO.^33^ On the contrary, standard markers associated with increased thrombosis risk were more prevalent among patients without a PFO. These findings suggest that if inherited or acquired thrombophilia contributes to PFO-associated strokes, it may not be detectable through routinely used thrombophilia panels.

In our study, initial thrombophilia testing and retesting appeared to affect treatment decisions, as anticoagulation was initiated more frequently in patients with baseline and persistent abnormal thrombophilia results. Based on our and others’ observations, targeted retesting focusing on specific thrombophilia markers rather than performing a full thrombophilia panel may reduce health care costs.^14^ However, the cost-effectiveness of this strategy should be further investigated in different health care settings.^34^

Key strengths of our study include its multicenter nature involving 19 European centers across 13 countries, which enhances its generalizability across diverse health care systems. Comprehensive thrombophilia testing strengthens the validity of the findings although there was variation between the centers with regard to composition of the coagulopathy panels. Furthermore, sensitivity analysis showed that associations between physical inactivity, history of VTE, low hemoglobin and HDL-cholesterol level, and abnormal thrombophilia findings remained robust after excluding patients with missing factor VIII and homocysteine values.

There are also some limitations to this study. The still relatively limited number of patients across 19 centers, together with the predominance of White European participants (94.4%), may limit the statistical power and generalizability of the results to other ethnic groups. While all patients included in our study underwent baseline thrombophilia testing and the protocol instructed on thrombophilia testing, there remained heterogeneity in the baseline testing across the sites. Carrying out retesting was at the discretion of local physicians, which introduces heterogeneity in the assessment of retesting results. In addition, 6.8% of the patients with abnormal thrombophilia results were anticoagulated at the time of baseline testing, which may have affected the results and interpretation of some markers. Although we adjusted for multiple confounders in regression analyses, residual confounding cannot be entirely excluded. Furthermore, missing data and differences in estrogen levels, including prior use of estrogen, may have influenced our findings. Last, our study focused on the prevalence and classification of thrombophilia abnormalities in young patients with stroke. Besides anticoagulation initiation, no other clinical outcomes, such as stroke recurrence or functional status, were investigated. Future studies should build on our findings by exploring, for example, potential hormonal impacts and prognostic and therapeutic implications of thrombophilia screening, which may enhance risk stratification in this patient group and define the optimal role of screening in clinical practice.

## Conclusions

Deviations in the thrombophilia panel appeared in over one-third of young patients with CIS although high-risk abnormalities were uncommon (5.2%). Targeted thrombophilia screening may be most relevant in patients with a history of VTE, physical inactivity, anemia, or low HDL-cholesterol. Systematic retesting appears necessary to capture persistent abnormalities. Current thrombophilia panels, largely based on VTE research, may not fully capture the spectrum of prothrombotic states in young-onset CIS, warranting further investigation into novel risk markers.

## ARTICLE INFORMATION

### Acknowledgments

The authors gratefully acknowledge Anu Eräkanto for her substantial technical assistance, the study nurses for their support, and all participants for their volunteer participation in the study. In addition, the authors are indebted to the clinical and research personnel at all participating sites.

### Sources of Funding

### Disclosures

Drs Tomppo, Jäkälä, and Huhtakangas reported grants from the Academy of Finland. Dr Tulkki reported grants from Suomen Lääketieteen Säätiö and Finska Läkaresällskapet. Dr Fonseca reported travel support from Bayer Healthcare and compensation from Bayer Healthcare for other services. Dr Ten Cate reported stock holdings in CoagulationProfile. The other authors report no conflicts.

### Supplemental Material

Tables S1–S11

Figures S1–S5

STROBE Checklist

## Supplementary Material


