# Supplementary material for: Thrombophilia Screening in Young Patients With Cryptogenic Ischemic Stroke
Source: Stroke. 2026 Mar 16;57(5):1242–55. doi: 10.1161/STROKEAHA.125.053251 (PMC13117596; doi:10.1161/STROKEAHA.125.053251)

SUPPLEMENTAL MATERIAL

Supplemental Table S1. Definitions of all comorbidities.

Variable	Definition
Abdominal obesity	Waist-to-hip ratio >0.85 in women and >0.90 in men, measured at study visit.
Current estrogen use	Any pre-stroke estrogen preparate use within a month prior to stroke onset, any route of administration. Patient history and medical records.
Current illicit drug use	Any illicit drug use within the past 12 months. Structured questionnaire.
Current smoking	Smoking at least one cigarette per day on average. Structured questionnaire.
Depression	Feeling depressed at 2 weeks in the prior 12 months. Structured questionnaire.
Diabetes mellitus	Prior diagnosis of any diabetes and/or prior antidiabetic medication. Patient history and medical records.
Dyslipidemia	Combination of either high total cholesterol, high LDL cholesterol, high triglycerides, low HDL cholesterol or use of antilipemic medication. Patient history and medical records.
Heavy alcohol consumption	>7 (women) and >14 (men) units per week or at least an average of two times per month ≥5 (women) and ≥7 (men) units per instance (binge drinking). Adaptation of the WHO Alcohol, Smoking and Substance Involvement Screening Test questionnaire.
Autoimmune disease	History of any auto-immune disease, excluding inflammatory bowel disease. Patient history and medical records.
Cardiovascular disease	History of coronary heart disease, congestive heart failure, peripheral arterial disease, or atrial fibrillation. Patient history and medical records.
Chronic kidney disease	Patient history and medical records.
Chronic liver disease	Patient history and medical records.
Chronic multisystem disorder	History of any of inflammatory bowel disease, autoimmune disease, chronic kidney/liver disease, or hematologic disease/thrombophilia. Patient history and medical records.
Inflammatory bowel disease	Patient history and medical records.
Hematologic disease	History of hematologic disease (excluding iron deficiency anemia) or known thrombophilia. Patient history and medical records.
History of venous thrombosis	Patient history and medical records.
Hypertension	Prior diagnosis of hypertension, prior antihypertensive medication or a mean of two office blood pressure measures ≥140/90 mmHg at study visit.

Low level of education	Either primary or lower secondary education, or upper secondary education
History of malignancy	History of any malignancy. Patient history and medical records.
Migraine with aura	Based on a validated migraine screener.
Obstructive sleep apnea syndrome	Patient history and medical records.
Perinatal conditions	Any of history of pregnancy diabetes, pregnancy hypertension of pre-eclampsia, eclampsia, or HELLP syndrome or currently being pregnant or in puerperium (90 days from end of pregnancy). Patient history and medical records.
Physical inactivity	Failing to meet the criteria for at least 1,500 metabolic equivalents per week. The short form of the International Physical Activity Questionnaire.
Psychosocial stress	At least several periods of stress at work or home (financial stress not considered). Structured questionnaire.
Unhealthy diet	A modified version of the Mediterranean Diet Score questionnaire. Higher scores represent healthier diet; a cutoff of 24 points defined unhealthy diet.

Table S2. Reference values for standard and thrombophilia laboratory markers used in the study in alphabetic order.

Laboratory characteristic	Normal reference values	Abnormal reference values for thrombophilia markers used in analysis
**Standard markers**		
Alanine aminotransferase (U/L)	<50 men, <35 women	
C-reactive protein (g/L)	<10	
Gamma-glutamyl transferase (U/L)	<60 men; <40 for women	
Hemoglobin (g/L)	134-167 for men, 117-155 for women	
High-density lipoprotein cholesterol (mmol/L)	>0.9	Low <0.9
Leukocyte count (E9/L)	3.4-8.2	
Low-density lipoprotein cholesterol (mmol/L)	<3.0	
Platelet count (E9/L)	150-360	
Prothrombin time (%)	70-130	
Thrombin clotting time (seconds)	16-19	
Total cholesterol (mmol/L)	<5.0	
Triglycerides (mmol/L)	<2.0	
**Thrombophilia markers**		
Anti-β2-glycoprotein antibodies (GPL)	<10	Low risk 10-40; High risk >40
Anticardiolipin antibodies (GPL)	<10	Low risk 10-40; High risk >40
Antithrombin activity (%)	>80	Low risk 60-79; High risk <60
Factor II 20210 gene mutation	Negative	Low risk, heterozygous; High risk, homozygous
Factor V Leiden gene mutation	Negative	Low risk, heterozygous; High risk, homozygous

Factor VIII activity (%)	70-150	Low risk 150-189; High risk >190
Free protein S antigen (%)	>70 men, >56 women	Low risk 40-70 men, 40-56 women; High risk <40
Homocysteine concentration (μmol/L)	0-15	Low risk 16-100; High risk >100
Lupus anticoagulant	Negative	High risk, positive
Protein C activity (%)	>70	Low risk 60-69; High risk <60

Table S3. Abnormalities in thrombophilia panel and clinical thrombosis risk categories, stratified by sex (n=556).

**Characteristic** (n missing)	**Overall (n=556)**	**Male (n=304)**	**Female (n=252)**	***P***
**Abnormality in thrombophilia panel**				
Any abnormality at baseline (0)	206 (37.1)	116 (38.2)	90 (35.7)	0.553
Any abnormality by 3 months (0)	47 (8.5)	24 (7.9)	23 (9.1)	0.603
Any abnormality by 12 months (0)	19 (3.4)	11 (3.6)	8 (3.2)	0.774
Persistent abnormality	45 (8.1)	24 (7.9)	21 (8.3)	0.850
**Screening performance**				
Thrombophilia panel tested at baseline (0)	556 (100)	304 (100)	252 (100)	NA
Thrombophilia panel tested at 3 months (0)	120 (21.6)	63 (20.7)	57 (22.6)	0.589
Thrombophilia panel tested at 12 months (0)	36 (6.5)	20 (6.6)	16 (6.3)	0.913
**Clinical thrombosis risk category at baseline (0)**				
Lowest risk	349 (62.8)	187 (61.5)	162 (64.3)	0.501
Low risk	161 (29.0)	92 (30.3)	69 (27.4)	0.456
High risk	29 (5.2)	19 (6.3)	10 (4.0)	0.228
Combined risk	17 (3.1)	6 (2.0)	11 (4.4)	0.103
**Clinical thrombosis risk category at 3 months (242/197)**				
Lowest risk	69 (59.0)	37 (59.7)	32 (58.2)	0.870
Low risk	33 (28.2)	19 (30.6)	14 (25.5)	0.533
High risk	13 (11.1)	6 (9.7)	7 (12.7)	0.600
Combined risk	2 (1.7)	0	2 (3.6)	0.130
**Clinical thrombosis risk category at 12 months (284/236)**				
Lowest risk	18 (50.0)	9 (45.0)	9 (56.3)	0.502

Low risk	13 (36.1)	8 (40.0)	5 (31.3)	0.587
High risk	5 (13.9)	3 (15.0)	2 (12.5)	0.829

Data are median (interquartile range) or mean (95% confidence interval) for continuous and n (%) for categorical variables. NA, not applicable.

Table S4. Thrombophilia results of young cryptogenic ischemic stroke patients, stratified by the persistency of abnormality in the thrombophilia panel at baseline and at 3 months or at 12 months (n=556).

**Characteristic** (n missing)	**No persistency (n=511)**	**Persistency (n=45)**	***P***
**Standard laboratory results**			
CRP level, g/L (69/0)	6.0 (8.6)	5.0 (0.0)	0.445
High (>10 g/L)	24 (5.1)	0	0.139
Hemoglobin, g/L (2/1)	142.1 (16.6)	138.2 (20.6)	0.142
Low (men <134 g/L, women <117 g/L)	43 (8.4)	4 (9.1)	0.883
Platelet count, E9/L (33/4)	254.2 (65.8)	249.3 (68.2)	0.648
High (>360 E9/L)	30 (6.3)	3 (7.3)	0.793
Leukocyte count, E9/L (16/3)	7.2 (2.2)	7.3 (2.4)	0.866
High (>8.2 E9/L)	206 (41.6)	18 (42.9)	0.876
Total cholesterol, mmol/L (7/0)	4.6 (1.0)	4.6 (1.2)	0.813
High (≥5.0 mmol/L)	182 (36.1)	12 (26.7)	0.204
LDL cholesterol, mmol/L (7/0)	3.0 (0.9)	2.9 (1.1)	0.408
High (≥3.0 mmol/L)	256 (50.8)	18 (40.0)	0.165
HDL cholesterol, mmol/L (8/0)	1.3 (0.4)	1.3 (0.3)	0.993
Low (<0.9 mmol/L)	178 (35.4)	14 (31.1)	0.565
Triglycerides, mmol/L(6/0)	1.3 (0.9)	1.4 (0.9)	0.562
High (≥2.0 mmol/L)	59 (11.7)	8 (17.8)	0.231
ALAT, U/L (63/5)	22.0 (15.0-32.0)	19.5 (14.0-27.8)	0.188
High (males >50 U/L, females >35 U/L)	55 (12.3)	2 (5.0)	0.170
GGT, U/L (111/4)	22.0 (14.0-36.0)	19.0 (13.0-27.0)	0.159
High (men >60 U/L, women >40 U/L)	45 (11.3)	6 (14.6)	0.519

Creatinine, µmol/L (6/2)	73.7 (16.0)	73.5 (19.5)	0.927
Glucose, mmol/L (48/3)	6.1 (1.9)	6.2 (2.2)	0.622
INR (64/6)	1.0 (1.0-1.1)	1.0 (1.0-1.1)	0.654
**Global coagulation tests**			
Prothrombin time, % (166/9)	88.8 (23.5)	90.6 (31.2)	0.674
Short (<70%)	39 (11.3)	5 (13.9)	0.644
Thrombin clotting time, sec (214/12)	19.9 (10.8)	18.7 (2.7)	0.534
Short (<16 sec)	39 (13.1)	2 (6.1)	0.243
**Inherited thrombophilia markers**			
Antithrombin activity, % (36/1)	103.5 (12.2)	101.2 (15.2)	0.255
Antithrombin activity risk category			<0.001
Normal (>80%)	465 (97.9)	39 (88.6)	
Low risk (60-79%)	9 (1.9)	5 (11.4)	
High risk (<60%)	1 (0.2)	0	
Protein C activity, % (25/1)	112.1 (22.5)	111.2 (28.2)	0.812
Protein C activity risk level			0.400
Normal (>70%)	478 (98.4)	42 (95.5)	
Low risk (60-69%)	4 (0.8)	1 (2.3)	
High risk (<60%)	4 (0.8)	1 (2.3)	
Free protein S antigen activity, % (30/1)	101.6 (26.4)	94.9 (30.1)	0.111
Free protein S antigen activity risk level			<0.001
Normal (men >70%, women >56%)	468 (97.3)	38 (86.4)	
Low risk (men 40-70%, women 40-56%)	10 (2.1)	5 (11.4)	
High risk (<40%)	3 (0.6)	1 (2.3)	
Factor II 20210 gene mutation (58/3)			0.011
Wildtype (normal)	450 (99.3)	40 (95.2)	

Heterozygous (low risk)	3 (0.7)	2 (4.8)	
Homozygous (high risk)	0	0	
Factor V Leiden mutation (56/2)			0.876
Wildtype (normal)	438 (96.3)	42 (97.7)	
Heterozygous (low risk)	16 (3.5)	1 (2.3)	
Homozygous (high risk)	1 (0.2)	0	
Factor VIII activity, % (259/10)	129.1 (51.9)	167.4 (57.5)	<0.001
Factor VIII activity risk level			<0.001
Normal (70-149%)	183 (76.3)	13 (38.2)	
Low risk (150-189%)	38 (15.8)	8 (23.5)	
High risk (>190%)	19 (7.9)	13 (38.2)	
Homocysteine concentration, μmol/L (116/4)	11.7 (5.9)	11.7 (7.4)	0.979
Normal (0-15 μmol/L)	326 (84.0)	34 (82.9)	0.856
Low risk (16-100 μmol/L)	62 (16.0)	7 (17.1)	
High risk (>100 μmol/L)	0	0	
**Acquired thrombophilia markers**			
Anticardiolipin antibodies (18/1)			<0.001
Negative, normal (<10 U/mL)	476 (96.6)	34 (77.3)	
Weakly positive, low risk (10-40 U/mL)	15 (3.0)	6 (13.6)	
Positive, high risk (>40 U/mL)	2 (0.4)	4 (9.1)	
Anti-β2-glycoprotein antibodies (26/0)			<0.001
Negative, normal (<10 U/mL)	480 (99.0)	37 (82.2)	
Weakly positive, low risk (10-40 U/mL)	4 (0.8)	5 (11.1)	
Positive, high risk (>40 U/mL)	1 (0.2)	3 (6.7)	
Lupus anticoagulant (20/0)			0.053
Negative, normal	481 (98.0)	42 (93.3)	

Positive, high risk	10 (2.0)	3 (6.7)	
Positive antiphospholipids (0)			
Single	24 (4.7)	12 (26.7)	<0.001
Double	1 (0.2)	3 (6.7)	<0.001
Triple	2 (0.4)	1 (2.2)	
**Overall thrombosis risk category (0)**			
Lowest risk	348 (68.1)	1 (2.2)	<0.001
Low risk	136 (26.6)	25 (55.6)	<0.001
High risk	16 (3.1)	13 (28.9)	<0.001

Data are median (interquartile range) or mean (95% confidence interval) for continuous and n (%) for categorical variables. NA, not applicable. CRP, C-reactive protein; LDL, Low-density lipoprotein; HDL, High-density lipoprotein; ALAT, Alanine aminotransferase; GGT Gamma-glutamyl transferase; INR, International normalized ratio; Positive antiphospholipids, presence of anticardiolipin antibodies or anti-b2-glycoprotein antibodies or lupus anticoagulant.

Table S5. Head-to-head comparisons of clinical characteristics at baseline between patients with lowest, low and high thrombosis risk (n=556).

	**Lowest risk vs. Low-risk**			**Lowest risk vs. High-risk**			**Low-risk vs. High-risk**
**Characteristic** (n missing)	**Lowest risk (n=349)**	**Low-risk (n=161)**	***P***	**Lowest risk (n=349)**	**High-risk (n=29)**	***P***	***P***
**Demographics**							
Age, years (0)	40.2 (33.3-45.2)	42.3 (37.0-46.9)	0.008	40.2 (33.3-45.2)	44.4 (35.1-47.3)	0.033	0.385
18-39 years	171 (49.0)	64 (39.8)	0.052	171 (49.0)	8 (27.6)	0.026	0.214
40-49 years	178 (51.0)	97 (60.2)		178 (51.0)	21 (72.4)		
Male sex (0)	187 (53.6)	92 (57.1)	0.453	187 (53.6)	19 (65.5)	0.215	0.400
White European race-ethnicity (0)	336 (96.3)	145 (90.1)	0.005	336 (96.3)	28 (96.6)	0.940	0.260
Low level of education (3)	195 (56.0)	89 (55.6)	0.931	195 (56.0)	14 (50.0)	0.536	0.581
**Family history**							
Stroke (120)	165 (52.4)	68 (56.2)	0.474	165 (52.4)	14 (53.8)	0.886	0.827
Stroke or venous thrombosis (120)	190 (60.3)	77 (63.6)	0.524	190 (60.3)	16 (61.5)	0.903	0.840
Ischemic heart disease (122)	175 (55.9)	70 (57.9)	0.715	175 (55.9)	19 (73.1)	0.089	0.150
Any family history (120)	250 (79.4)	95 (78.5)	0.844	250 (79.4)	25 (96.2)	0.037	0.035
**Patent foramen ovale (PFO)**							
High-risk PFO (19)	131 (38.9)	61 (39.1)	0.961	131 (38.9)	11 (39.3)	0.966	0.985
**Traditional risk factors**							
Cardiovascular disease (0)	8 (2.3)	8 (5.0)	0.107	8 (2.3)	0	0.410	0.220
Diabetes mellitus (0)	9 (2.6)	9 (5.6)	0.087	9 (2.6)	0	0.381	0.192

Dyslipidemia (0)	197 (56.4)	104 (64.6)	0.082	197 (56.4)	16 (55.2)	0.894	0.333
Hypertension (0)	121 (34.7)	58 (36.0)	0.766	121 (34.7)	13 (44.8)	0.272	0.367
Obstructive sleep apnea (4)	11 (3.2)	0	0.022	11 (3.2)	0	0.338	NA
Abdominal obesity (2)	214 (61.3)	88 (55.3)	0.204	214 (61.3)	20 (69.0)	0.415	0.172
Current smoking (3)	114 (32.9)	47 (29.4)	0.434	114 (32.9)	9 (31.0)	0.841	0.857
Heavy alcohol use (1)	46 (13.2)	19 (11.9)	0.682	46 (13.2)	3 (10.3)	0.662	0.813
Unhealthy diet (5)	176 (50.9)	75 (47.2)	0.440	176 (50.9)	18 (62.1)	0.246	0.140
Physical inactivity (7)	89 (25.9)	65 (40.6)	<0.001	89 (25.9)	13 (44.8)	0.029	0.672
Psychosocial stress (1)	172 (49.4)	66 (41.0)	0.076	172 (49.4)	13 (44.8)	0.634	0.700
Depression (1)	102 (29.3)	45 (28.0)	0.753	102 (29.3)	9 (31.0)	0.845	0.735
No. of traditional risk factors (18)	3 (2-4)	3 (2-4)	0.594	3 (2-4)	4 (2-5)	0.585	0.461
**Non-traditional risk factors**							
History of chronic multisystem disorder (0)	43 (12.3)	31 (19.3)	0.039	43 (12.3)	3 (10.3)	0.754	0.249
History of venous thromboembolism (2)	7 (2.0)	9 (5.6)	0.032	7 (2.0)	2 (6.9)	0.099	0.782
History of malignancy (0)	5 (1.4)	5 (3.1)	0.205	5 (1.4)	1 (3.4)	0.404	0.923
History of migraine with aura (1)	142 (40.8)	52 (32.3)	0.066	142 (40.8)	16 (55.2)	0.132	0.018
Illicit drug use within the past 12 months (0)	26 (7.4)	9 (5.6)	0.440	26 (7.4)	2 (6.9)	0.913	0.782
No. of non-traditional risk factors (3)	1 (0-1)	0 (0-1)	0.399	1 (0-1)	1 (0-1)	0.003	0.004
**Female-sex-specific risk factors**							

Perinatal condition (6)	22 (13.9)	7 (10.4)	0.477	22 (13.9)	4 (40.0)	0.027	0.013
Current estrogen use (0)	43 (26.5)	20 (29.0)	0.703	43 (26.5)	3 (30.0)	0.811	0.947
One or more female-sex-specific risk factor (7)	60 (38.2)	26 (38.8)	0.934	60 (38.2)	6 (60.0)	0.172	0.205
**Medication and infections at the time of coagulopathy testing**							
Antiplatelet (5)	242 (70.1)	116 (72.5)	0.588	242 (70.1)	19 (65.5)	0.602	0.444
Anticoagulation (5)	41 (11.9)	11 (6.9)	0.085	41 (11.9)	2 (6.9)	0.419	0.997
Infection symptoms at time of enrollment (10)	18 (5.3)	15 (9.4)	0.080	18 (5.3)	1 (3.4)	0.670	0.288
**NIH Stroke Scale**							
Admission NIHSS (2)	2 (0-4)	2 (0-4)	0.625	2 (0-4)	1 (0-4)	0.412	0.573
NIHSS categories (2)			0.791			0.475	0.673
NIHSS 0	87 (25.0)	44 (27.3)		87 (25.0)	9 (31.0)		
NIHSS 1-4	176 (50.6)	82 (50.9)		176 (50.6)	13 (44.8)		
NIHSS 5-9	50 (14.4)	23 (14.3)		50 (14.4)	6 (20.7)		
NIHSS ≥10	35 (10.1)	12 (7.5)		35 (10.1)	1 (3.4)		
**Standard laboratory results**							
CRP level, g/L (43)	5.6 (2.7)	7.1 (15.2)	0.240	5.6 (2.7)	5.0 (0)	<0.001	0.461
High (>10 g/L)	17 (5.1)	6 (4.3)	0.719	17 (5.1)	0	0.211	0.591
Admission Hemoglobin, g/L (3)	142.9 (14.8)	142.0 (16.9)	0.535	142.9 (14.8)	135.4 (27.9)	0.164	0.087
Low (men <134 g/L, women <117 g/L)	22 (6.3)	17 (10.7)	0.087	22 (6.3)	4 (13.8)	0.127	0.626
Platelet count, E9/L (37)	255.1 (61.8)	249.6 (69.8)	0.392	255.1 (61.8)	257.5 (67.0)	0.847	0.588

High (>360 E9/L)	18 (5.5)	11 (7.4)	0.416	18 (5.5)	2 (7.4)	0.681	0.996
Leukocyte count, E9/L (19)	8.2 (2.7)	7.9 (2.8)	0.298	8.2 (2.7)	8.5 (2.4)	0.517	0.275
High (>8.2 E9/L)	140 (41.7)	59 (38.1)	0.450	140 (41.7)	14 (48.3)	0.489	0.302
Total cholesterol, mmol/L (7)	4.6 (1.0)	4.7 (1.1)	0.338	4.6 (1.0)	4.6 (1.1)	0.833	0.545
High (≥5.0 mmol/L)	116 (33.5)	61 (38.9)	0.246	116 (33.5)	9 (31.0)	0.785	0.425
LDL cholesterol, mmol/L (7)							
High (≥3.0 mmol/L)	167 (48.3)	83 (52.9)	0.339	167 (48.3)	14 (48.3)	0.999	0.649
HDL cholesterol, mmol/L (8)							
Low HDL cholesterol (<0.9 mmol/L)	113 (32.8)	63 (39.9)	0.126	113 (32.8)	11 (37.9)	0.577	0.844
Triglycerides, mmol/L (6)							
High (≥2.0 mmol/L)	40 (11.6)	21 (13.3)	0.581	40 (11.6)	1 (3.4)	0.179	0.130
ALAT, U/L (68)	22.0 (15.0-32.0)	21.0 (14.0-33.0)	0.712	22.0 (15.0-32.0)	22.0 (19.0-31.8)	0.277	0.182
High (men >50 U/L, women >35 U/L)	39 (12.3)	13 (10.1)	0.500	39 (12.3)	4 (14.3)	0.766	0.516
GGT, U/L (115)	20.0 (14.0-32.8)	22.0 (14.0-37.3)	0.166	20.0 (14.0-32.8)	26.5 (16.0-43.0)	0.094	0.338
High (men >60 U/L, women >40 U/L)	27 (10.2)	16 (11.9)	0.603	27 (10.2)	6 (23.1)	0.049	0.131
Creatinine, µmol/L (8)	73.1 (16.3)	75.4 (16.3)	0.144	73.1 (16.3)	73.8 (12.6)	0.812	0.636
Glucose, mmol/L (51)	6.1 (1.9)	5.9 (1.5)	0.487	6.1 (1.9)	6.4 (1.7)	0.377	0.150
INR (70)	1.0 (1.0-1.1)	1.0 (1.0-1.0)	0.116	1.0 (1.0-1.1)	1.0 (1.1-1)	0.635	0.204
**Global coagulation tests**							
Prothrombin time, % (175)	87.8 (22.3)	90.0 (26.2)	0.424	87.8 (22.3)	94.4 (23.4)	0.196	0.482
Short (<70%)	27 (10.7)	13 (13.3)	0.493	27 (10.7)	2 (9.5)	0.869	0.639
Thrombin clotting time, sec (226)	20.2 (11.1)	19.3 (10.1)	0.462	20.2 (11.1)	19.0 (3.2)	0.630	0.922
Short (<16 sec)	20 (10.5)	15 (13.9)	0.386	20 (10.5)	2 (9.5)	0.887	0.588

Data are median (interquartile range) or mean (95% confidence interval) for continuous and n (%) for categorical variables. NA, not applicable. NIHSS, National insmatitutes of health stroke scale; CRP, C-reactive protein; LDL, Low-density lipoprotein; HDL, High-density lipoprotein; Dyslipidemia, A history of hypercholesterolemia in combination with high total cholesterol and high LDL and high triglycerides and low HDL; ALAT, Alanine aminotransferase; GGT Gamma-glutamyl transferase; INR, International normalized ratio.

Table S6. Odds ratios and 95% confidence intervals (CIs) from multivariable logistic regression models assessing clinical and laboratory variables associated with the secondary outcome measure, high-risk findings in the thrombophilia panel.

**Predictor**	**Model 1: Adjusted for age**		**Model 2: Adjusted age and clinical risk factors**		**Model 3: Adjusted for age, clinical factors and laboratory values**	
	**OR (95% CI)**	***P***	**OR (95% CI)**	***P***	**OR (95% CI)**	***P***
Age (40-49 vs 18-39 years)	2.23 (0.97-5.12)	0.059	2.24 (0.97-5.14)	0.058	-	-
Migraine with aura	-	-	-	-	0.34 (0.08-1.48)	0.150
High GGT level (men >60 U/L, women >40 U/L)	-	-	-	-	2.45 (0.93-6.45)	0.069

Data are in odds ratios (OR) and 95% confidence interval. Variables were retained based on backward stepwise selection (likelihood ratio), with a significance level for removal set at p >0.10.

Table S7. Odds ratios (ORs) and 95% confidence intervals (CIs) from multivariable logistic regression models for primary outcome measure, any abnormality in the thrombophilia panel among men.

**Predictor**	**Model 1: Adjusted demographics (age, sex, race)**		**Model 2: Adjusted demographics and clinical risk factors**		**Model 3: Adjusted for demographics, clinical factors and laboratory values**	
	**OR (95% CI)**	***P***	**OR (95% CI)**	***P***	**OR (95% CI)**	***P***
Age (40-49 vs 18-39 years)	1.04 (1.01-1.08)	0.014	1.03 (1.00-1.07)	0.064	1.64 (0.98-2.76)	0.060
Race (white European vs other)	2.60 (0.91-7.44)	0.075	-	-	-	-
Physical inactivity	-	-	1.99 (1.19-3.33)	0.009	2.05 (1.22-3.46)	0.007
History of chronic multisystem disorder	-	-	1.96 (0.92-4.19)	0.083	2.02 (0.94-4.36)	0.072
Low Hemoglobin (M <134 g/L, F <117 g/L)	-	-	-	-	2.40 (0.96-6.01)	0.061

Data are in odds ratios (OR) and 95% confidence interval. Variables were retained based on backward stepwise selection (likelihood ratio), with a significance level for removal set at p > 0.10.

Table S8. Odds ratios (ORs) and 95% confidence intervals (CIs) from multivariable logistic regression models for primary outcome measure, any abnormality in the thrombophilia panel among women.

**Predictor**	**Model 1: Adjusted demographics (age, sex, race)**		**Model 2: Adjusted demographics and clinical risk factors**		**Model 3: Adjusted for demographics, clinical factors and laboratory values**	
	**OR (95% CI)**	***P***	**OR (95% CI)**	***P***	**OR (95% CI)**	***P***
Age (40-49 vs 18-39 years)	1.02 (0.99-1.05)	0.223	-	-	-	-
Race (white European vs other)	2.28 (0.79-6.55)	0.127	-	-	-	-
Physical inactivity	-	-	2.13 (1.22-3.71)	0.008	2.18 (1.25-3.83)	0.006
History of venous thromboembolism	-	-	5.08 (1.26-20.55)	0.023	3.74 (0.86-16.37)	0.080

Data are in odds ratios (OR) and 95% confidence interval. Variables were retained based on backward stepwise selection (likelihood ratio), with a significance level for removal set at $p > 0.10$.

Table S9. Thrombophilia results of young cryptogenic ischemic stroke patients, stratified by the presence of a clinically relevant high-risk patent foramen ovale (PFO) (n=537).

**Characteristic** (n missing no PFO/High-risk PFO)	**No PFO (n=330)**	**High-risk PFO (n=207)**	***P***
**Standard laboratory results**			
CRP level, g/L (29/12)	5 (5-12)	5 (5-5)	0.026
High (>10 g/L)	18 (6.0)	4 (2.1)	0.037
Hemoglobin, g/L (2/1)	142.1 (18.4)	141.1 (13.5)	0.499
Low (men <134 g/L, women <117 g/L)	35 (10.7)	10 (4.9)	0.019
Platelet count, E9/L (18/17)	256.7 (71.0)	250.5 (56.6)	0.281
High (>360 E9/L)	27 (8.7)	6 (3.2)	0.016
Leukocyte count, E9/L (11/7)	8.6 (2.9)	7.4 (2.3)	<0.001
High (>8.2 E9/L)	157 (49.2)	58 (29.0)	<0.001
Total cholesterol, mmol/L (4/2)	4.7 (1.0)	4.6 (0.9)	0.335
High (≥5.0 mmol/L)	121 (37.1)	67 (32.7)	0.298
LDL cholesterol, mmol/L (5/1)	3.0 (1.0)	3.0 (0.8)	0.305
High (≥3.0 mmol/L)	170 (52.3)	98 (47.6)	0.288
HDL cholesterol, mmol/L	1.3 (0.4)	1.4 (0.4)	0.010
Low (<0.9 mmol/L)	36 (11.1)	15 (7.3)	0.153
Triglycerides, mmol/L	1.4 (0.9)	1.3 (0.8)	0.275
High (≥2.0 mmol/L)	39 (12.0)	24 (11.6)	0.888
ALAT, U/L (40/25)	21.5 (15.0-34.0)	21.0 (15.0-29.0)	0.190
High (males >50 U/L, females >35 U/L)	42 (14.5)	13 (7.1)	0.016
GGT, U/L (82/26)	22.0 (15.0-37.0)	19.0 (13.0-29.0)	0.040
High (men >60 U/L, women >40 U/L)	30 (12.1)	19 (10.5)	0.607

Creatinine, µmol/L (4/3)	73.9 (16.4)	73.2 (15.8)	0.628
Glucose, mmol/L (38/11)	6.2 (1.9)	5.9 (1.7)	0.168
INR (31/38)	1.0 (1.0-1.1)	1.0 (1.0-1.1)	0.450
**Global coagulation tests**			
Prothrombin time, % (90/75)	88.4 (24.9)	90.8 (22.0)	0.357
Short (<70%)	29 (12.1)	13 (9.8)	0.650
Thrombin clotting time, sec (140/74)	19.9 (9.9)	18.8 (3.4)	0.245
Short (<16 sec)	22 (11.6)	16 (12.0)	0.704
**Inherited thrombophilia markers**			
Antithrombin activity, % (18/15)	102.9 (12.2)	103.7 (13.1)	0.464
Antithrombin activity risk category			0.436
Normal (>80%)	303 (97.1)	186 (96.9)	
Low risk (60-79%)	9 (2.9)	5 (2.6)	
High risk (<60%)	0	1 (0.5)	
Protein C activity, % (15/8)	112.3 (23.3)	111.1 (22.5)	0.578
Protein C activity risk level			0.203
Normal (>70%)	307 (97.5)	197 (99.0)	
Low risk (60-69%)	5 (1.6)	0	
High risk (<60%)	3 (1.0)	2 (1.0)	
Free protein S antigen activity, % (16/12)	102.9 (26.0)	97.8 (28.0)	0.034
Free protein S antigen activity risk level			0.301
Normal (men >70%, women >56%)	300 (95.5)	190 (97.4)	
Low risk (men 40-70%, women 40-56%)	12 (3.8)	3 (1.5)	
High risk (<40%)	2 (0.6)	2 (1.0)	
Factor II 20210 gene mutation (49/8)			0.653
Wildtype (normal)	279 (99.3)	196 (98.5)	

Heterozygous (low risk)	2 (0.7)	3 (1.5)	
Homozygous (high risk)	0	0	
Factor V Leiden gene mutation (48/7)			0.564
Wildtype (normal)	271 (96.1)	195 (97.5)	
Heterozygous (low risk)	10 (3.5)	5 (2.5)	
Homozygous (high risk)	1 (0.4)	0	
Factor VIII activity, % (177/76)	140.7 (55.2)	125.2 (51.1)	0.015
Factor VIII activity risk level			0.366
Normal (70-149%)	103 (68.2)	91 (75.8)	
Low risk (150-189%)	28 (18.5)	18 (15.0)	
High risk (>190%)	20 (13.2)	11 (9.2)	
Homocysteine concentration, µmol/L (89/26)	11.8 (6.3)	11.4 (5.7)	0.500
Normal (0-15 µmol/L)	194 (81.2)	149 (84.2)	0.425
Low risk (16-100 µmol/L)	45 (18.8)	28 (15.8)	
High risk (>100 µmol/L)	0	0	
**Acquired thrombophilia markers**			
Anticardiolipin antibodies (12/6)			0.240
Negative, normal (<10 U/mL)	298 (93.7)	195 (97.0)	
Weakly positive, low risk (10-40 U/mL)	16 (5.0)	5 (2.5)	
Positive, high risk (>40 U/mL)	4 (1.3)	1 (0.5)	
Anti-β2-glycoprotein antibodies (15/9)			0.715
Negative, normal (<10 U/mL)	307 (97.5)	195 (98.5)	
Weakly positive, low risk (10-40 U/mL)	6 (1.9)	2 (1.0)	
Positive, high risk (>40 U/mL)	2 (0.6)	1 (0.5)	
Lupus anticoagulant (9/9)			0.547
Negative, normal	314 (97.8)	192 (97.0)	

Positive, high risk	7 (2.2)	6 (3.0)	
Positive antiphospholipids (0)			
Single	22 (6.7)	13 (6.3)	0.860
Double	2 (0.6)	1 (0.5)	0.381
Triple	3 (0.9)	0	
**Overall thrombosis risk category (0)**			
Lowest risk	206 (62.4)	131 (63.3)	0.841
Low risk	95 (28.8)	61 (29.5)	0.866
High risk	17 (5.2)	11 (5.3)	0.934

Data are median (interquartile range) or mean (95% confidence interval) for continuous and n (%) for categorical variables. NA, not applicable. CRP, C-reactive protein; LDL, Low-density lipoprotein; HDL, High-density lipoprotein; ALAT, Alanine aminotransferase; GGT Gamma-glutamyl transferase; INR, International normalized ratio; Positive antiphospholipids, presence of anticardiolipin antibodies or anti-b2-glycoprotein antibodies or lupus anticoagulant.

Table S10. Thrombophilia results of young cryptogenic ischemic stroke patients, stratified by the use of anticoagulation by 12 months (n=556).

**Characteristic** (n missing)	**No anticoagulation use at 12 months (n=504)**	**Anticoagulation use at 12 months (n=52)**	***P***
**Abnormality in thrombophilia panel**			
Any abnormality at baseline (0)	180 (35.7)	26 (50.0)	0.042
Any abnormality by 3 months (0)	34 (6.7)	13 (25.0)	<0.001
Any abnormality by 12 months (0)	13 (2.6)	6 (11.5)	<0.001
Persistent abnormality (0)	31 (6.2)	14 (26.9)	<0.001
**Clinical thrombosis risk category at baseline (0)**			
Lowest risk	323 (64.1)	26 (50.0)	0.045
Low risk	145 (28.8)	16 (30.8)	0.762
High risk	23 (4.6)	6 (11.5)	0.031
Combined risk	13 (2.6)	4 (7.7)	0.041
**Clinical thrombosis risk category by 3 months (242/197)**			
Lowest risk	61 (63.5)	8 (38.1)	0.032
Low risk	25 (26.0)	8 (38.1)	0.266
High risk	10 (10.4)	3 (14.3)	0.609
Combined risk	0	2 (9.5)	0.031
**Clinical thrombosis risk category by 12 months (284/236)**			
Lowest risk	16 (55.2)	2 (28.6)	0.402
Low risk	9 (31.0)	4 (57.1)	0.225
High risk	4 (13.8)	1 (14.3)	1.000

Data are median (interquartile range) or mean (95% confidence interval) for continuous and n (%) for categorical variables. NA, not applicable.

Table S11. Odds ratios and 95% confidence intervals (CIs) from multivariable logistic regression models assessing clinical and laboratory variables associated with the primary outcome measure, any abnormal findings in the thrombophilia panel. Sensitivity analysis excluding patients with missing data on factor VIII activity and homocysteine concentration (n=245).

**Predictor**	**Model 1: Adjusted demographics (age, sex, race)**		**Model 2: Adjusted demographics and clinical risk factors**		**Model 3: Adjusted for demographics, clinical factors and laboratory values**	
	**OR (95% CI)**	***P***	**OR (95% CI)**	***P***	**OR (95% CI)**	***P***
Age (40-49 vs 18-39 years)	1.03 (1.00-1.07)	0.052	-	-	-	-
Race (white European vs other)	7.43 (0.89-62.02)	0.064	6.54 (0.76-56.25)	0.087	5.90 (0.68-51.22)	0.107
History of venous thromboembolism	-	-	3.60 (0.89-14.58)	0.072	3.28 (0.78-13.74)	0.104
Physical inactivity	-	-	3.10 (1.70-5.64)	0.000	2.81 (1.52-5.19)	0.001
Diabetes mellitus	-	-	7.55 (0.88-64.51)	0.065	7.11 (0.80-62.89)	0.078
Low Hemoglobin (men <134 g/L, women <117 g/L)	-	-	-	-	6.86 (1.47-31.99)	0.014
Low HDL cholesterol (<0.9 mmol/L)	-	-	-	-	1.19 (0.43-3.32)	0.735

Data are in odds ratios (OR) and 95% confidence interval. Variables were retained based on backward stepwise selection (likelihood ratio), with a significance level for removal set at p > 0.10. HDL, high-density lipoprotein.

Figure S1. Flowchart of data selection.

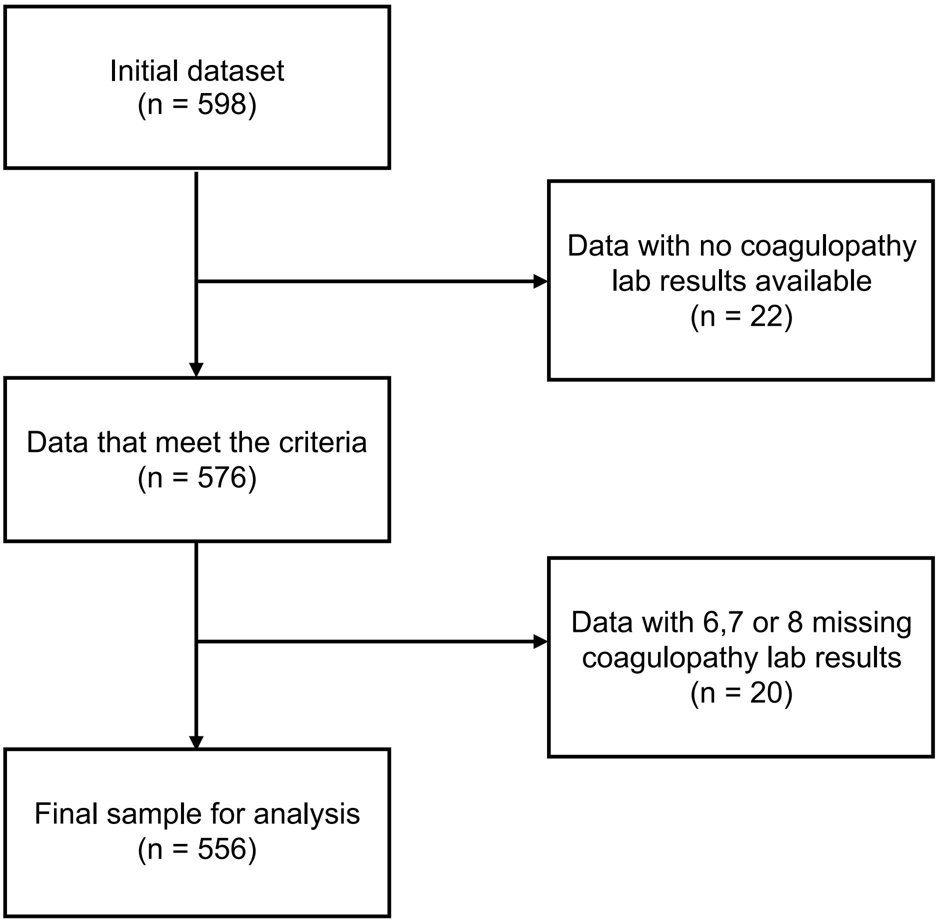

Figure S2. Descriptive analysis of continuous thrombophilia markers, stratified by sex (n=556).

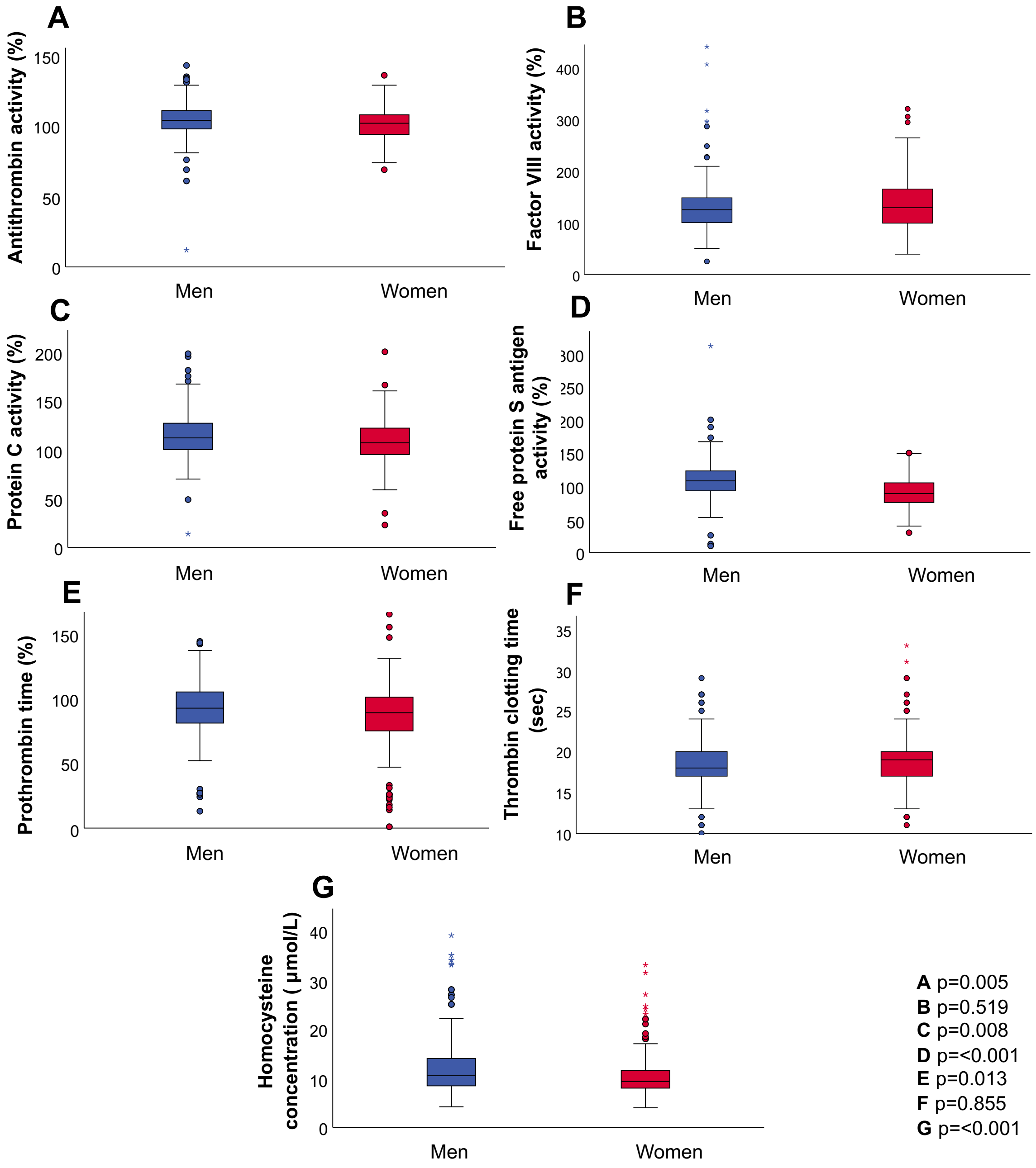

Figure S3. ROC curves with area under the curve for multivariable logistic regression models for primary outcome measure, any abnormal findings in the thrombophilia panel.

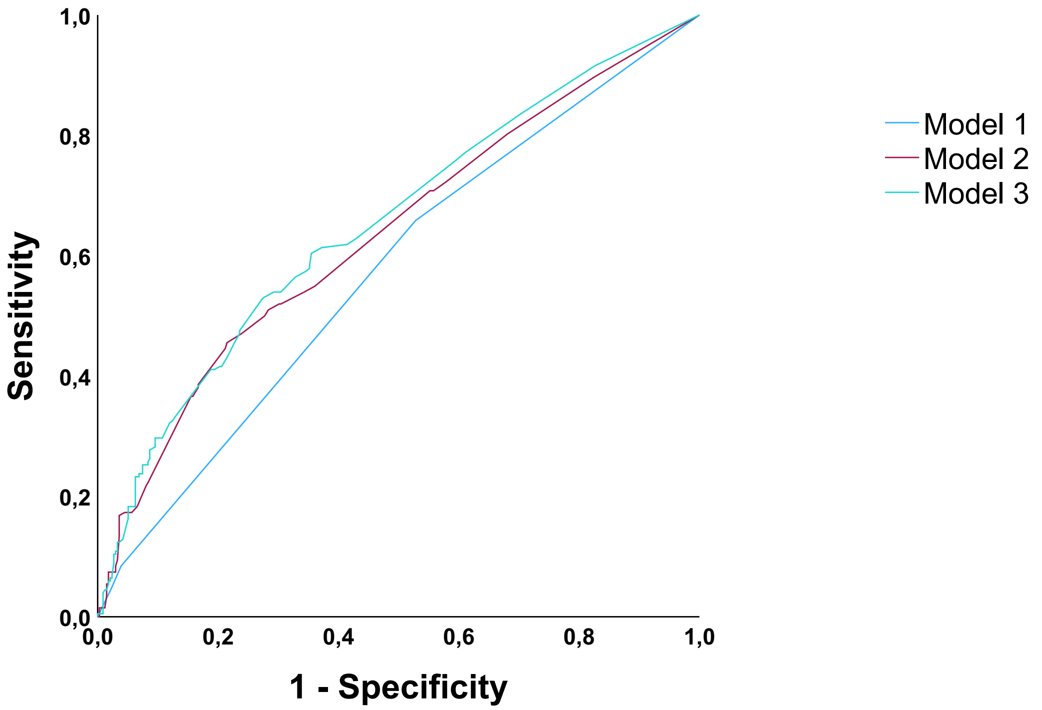

Figure S4. Descriptive analysis of continuous thrombophilia markers, stratified by the presence of clinically relevant patent foramen ovale (PFO) (n=537).

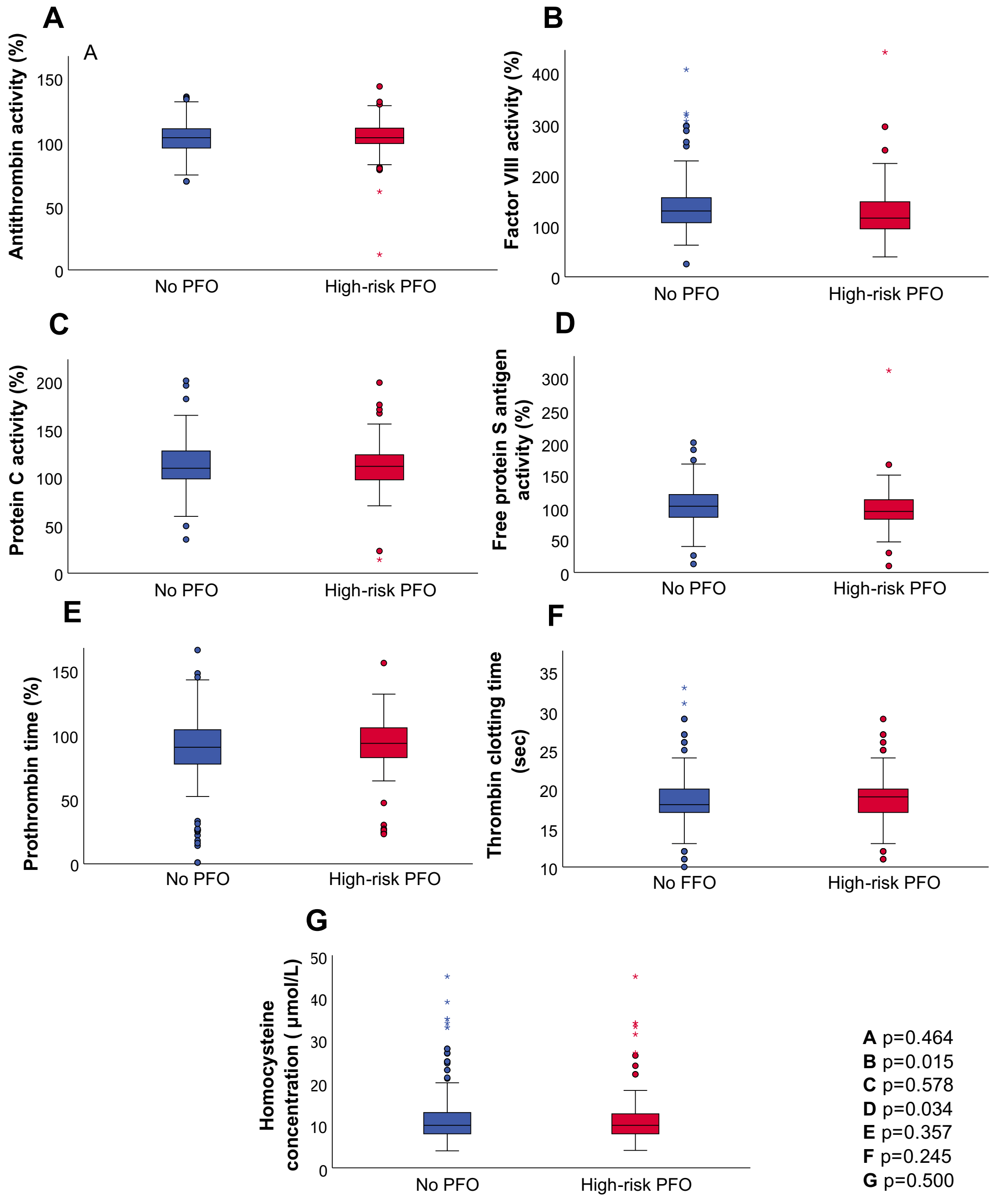

Figure S5. Sankey diagram illustrating change of initiation anticoagulation (AC) in patients with and with no coagulopathy (abnormal thrombophilia testing results), from baseline to three months and twelve months.

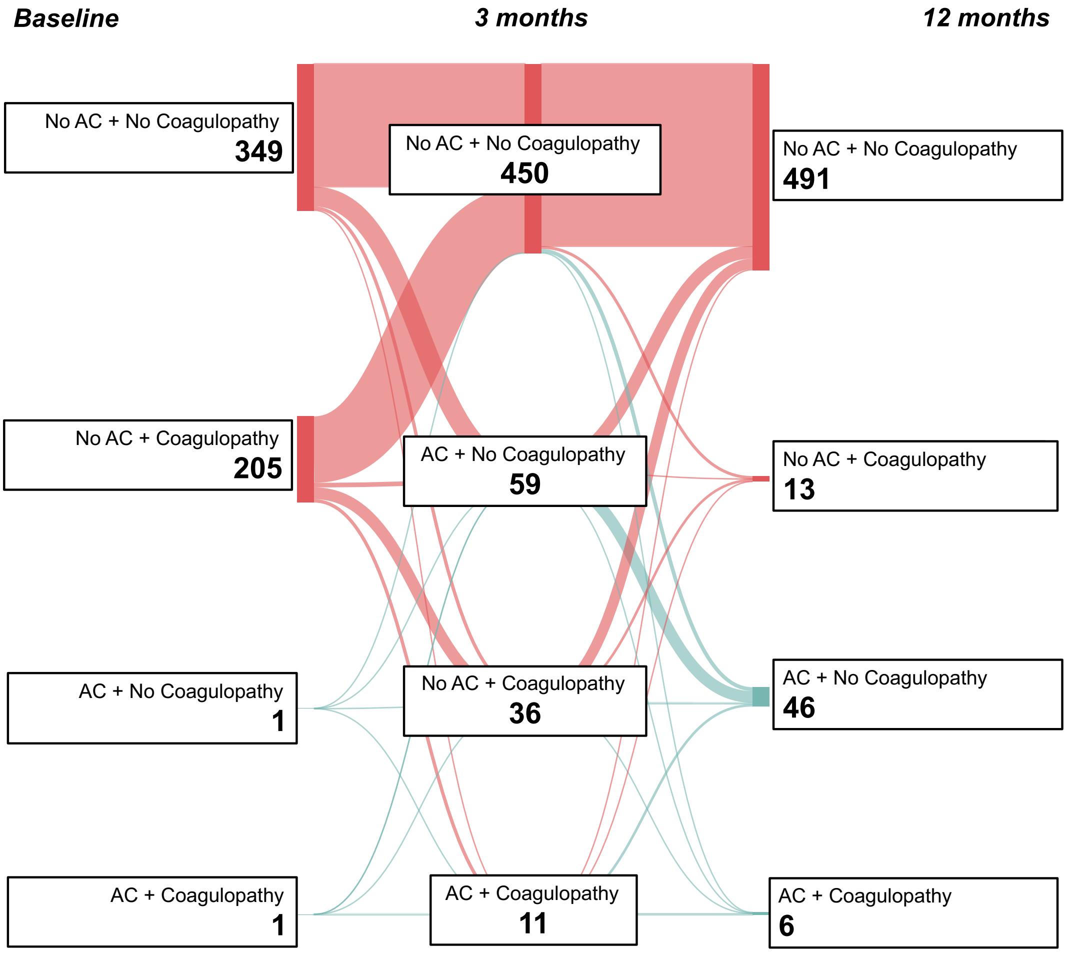

Supplement: Supplementary file 1 [file str-57-1242-s001.pdf]
